# Triglyceride glucose index - body mass index predicts insulin resistance, metabolic syndrome and associates with impaired ovulation in Chinese women with polycystic ovary syndrome

**DOI:** 10.3389/fendo.2025.1653636

**Published:** 2025-09-18

**Authors:** Muxin Guan, Jiaxing Feng, Mengyi Zhu, Yu Wang, Baichao Shi, Fengjuan Lu, Jiannan Yu, Zhuwei Gao, Hong Yu, Xiaoke Wu

**Affiliations:** ^1^ Heilongjiang University of Chinese Medicine, Harbin, China; ^2^ First Affiliated Hospital, Heilongjiang University of Chinese Medicine, Harbin, China; ^3^ The First School of Clinical Medicine, Zhejiang Chinese Medical University, Hangzhou, China

**Keywords:** polycystic ovary syndrome, triglyceride glucose index-body mass index, insulin resistance, metabolic syndrome, fertility outcomes

## Abstract

**Background:**

Insulin resistance (IR) and metabolic syndrome (MetS) are highly prevalent and pathophysiologically central features of polycystic ovary syndrome (PCOS). However, their assessment is challenged by the limitations of gold-standard diagnostic methods. The clinical utility of the novel triglyceride glucose index - body mass index (TyG-BMI) for predicting IR, MetS, and its association with fertility outcomes in Chinese women with PCOS remains unexplored and warrants investigation.

**Objective:**

To evaluate the association between TyG-BMI and IR and MetS, and fertility outcomes in women with PCOS.

**Methods:**

We used data of 855 participants of the Acupuncture and Clomiphene for Chinese Women with Polycystic Ovary Syndrome (PCOSAct) trial. Linear trend tests and logistic regression evaluated relationships between TyG-BMI and anthropometric, hormonal, metabolic, and fertility outcomes. Receiver operating characteristic (ROC) curves assessed TyG-BMI’s predictive value for IR and MetS. RCS analysis was used to examine threshold effects between TyG-BMI and IR, MetS, and ovulation. A likelihood ratio test was further incorporated to validate the model fit.

**Results:**

TyG-BMI was positively association with IR (OR: 2.747, 95% CI: 1.942–3.887) and MetS (OR: 4.176, 95% CI: 2.278–7.653). TyG-BMI had a strong predictive performance, with AUC_IR_ of 0.841 and AUC_MetS_ of 0.899. For fertility outcomes, after adjusting for confounders, only ovulation showed a significant negative association (OR: 0.984, 95% CI: 0.973–0.994). The study revealed significant nonlinear associations between TyG-BMI and both IR and MetS, but a linear link with ovulation status. The inflection point occurred at a TyG-BMI of 203. Below this, IR risk increased progressively with TyG-BMI, plateauing above it. Above 203, MetS prevalence continued to increase, while ovulation rates declined inversely.

**Conclusion:**

Elevated TyG-BMI is strongly associated with worsened IR and MetS in PCOS women, serving as a practical screening tool for these conditions, while also demonstrating a potential negative impact on ovulation.

## Introduction

Polycystic ovary syndrome (PCOS), the most prevalent endocrine disorder in reproductive-aged women, affects up to 15% of women worldwide, depending on diagnostic criteria (1, 2). It is clinically characterized by hyperandrogenism, oligo-ovulation, and polycystic ovarian morphology, with heterogeneous manifestations ranging from obesity, menstrual irregularities, and infertility to metabolic complications such as insulin resistance (IR), dyslipidemia, metabolic syndrome (MetS), and an increased risk of cardiovascular disease (CVD) (3).

IR is a key pathophysiological feature of PCOS, contributing to both endocrine dysfunction and metabolic disturbances (4). Epidemiological data indicate a wide prevalence range of IR (12%-60%) in women with PCOS, influenced by diagnostic criteria, assessment methods, and population characteristics (5). While the hyperinsulinemic-euglycemic clamp (HIEC) remains the gold standard for IR assessment (6), its clinical utility is limited by invasiveness, high cost, and technical complexity. The more commonly used homeostasis model assessment of insulin resistance (HOMA-IR) provides a practical alternative but has significant limitations, particularly in insulin-treated patients or those with β-cell dysfunction (7). The diagnostic challenges extend to MetS, where incidence rates vary substantially across ethnic groups and diagnostic criteria. Notably, metabolic dysregulation is highly prevalent in PCOS, with 54.9% of patients classified as overweight and 25.7% meeting MetS criteria (8). Importantly, Chinese women with PCOS demonstrate a distinct metabolic profile, showing higher rates of metabolic disorders but lower MetS prevalence when assessed by IDF criteria (9). Given the proven impact of weight control and metabolic improvement on reproductive outcomes in PCOS (10, 11), early identification of IR and MetS is critical. However, current diagnostic limitations underscore the urgent need for simple, reliable tools to stratify metabolic risk in this population.

The triglyceride-glucose index (TyG), calculated from fasting triglyceride and glucose levels, has been validated as a reliable surrogate marker for IR (12). It exhibits broader clinical significance, demonstrating an association with higher mortality risk in Cardiovascular-Kidney-Metabolic (CKM) syndrome stages 0-3 (13). Notably, TyG shows U-shaped correlations with all-cause and cardiovascular mortality, suggesting intervention thresholds at 9.104 and 8.758, respectively (14). Furthermore, elevated TyG status correlates with early clinical manifestations of pancreatic ductal adenocarcinoma (PDAC) (15), while emerging evidence links it to metabolic dysregulation in post-COVID-19 syndrome (16).

In PCOS populations, where obesity frequently coexists with metabolic disturbances such as glucose intolerance and IR (17, 18), integrating BMI may enhance diagnostic utility. BMI is a fundamental measure of obesity and indicates IR through its association with adipose tissue lipolysis and free fatty acid-mediated insulin sensitivity modulation.

This rationale underlies the proposed TyG-BMI composite index as a potentially superior IR marker (19). Recent studies indicate that TyG-BMI outperforms TyG alone in metabolic risk assessment. For instance, research in Korean adults demonstrated TyG-BMI’s superior predictive power for IR compared to other parameters (TyG, TyG-WC, and TyG-WHtR) (20). Further supporting this, another study incorporating additional adiposity indicators found TyG-BMI achieved the highest AUC (0.801) among all evaluated metrics (21). The clinical utility of TyG-BMI in PCOS, particularly regarding its diagnostic accuracy for IR and MetS and its association with fertility outcomes following ovulation induction, remains unexplored. Addressing these knowledge gaps could provide valuable insights for PCOS management, warranting systematic investigation.

This study had two primary objectives: firstly, to systematically examine the relationships between TyG-BMI and baseline characteristics, including anthropometric measures, metabolic profiles, and reproductive characteristics in infertile women with PCOS; secondly, to determine the diagnostic accuracy of TyG-BMI for predicting IR and MetS in this population.

## Patients and methods

### Participants

This secondary analysis utilized data from the PCOS Acupuncture and Clomiphene Trial (PCOSAct), a multicenter, randomized, double-blind controlled clinical trial (ClinicalTrials.gov NCT01573858; Chinese Clinical Trial Registry ChiCTR-TRC-12002081). The study enrolled 1,000 infertile women aged 20–40 years meeting the modified Rotterdam criteria (22) for PCOS across 27 hospitals in mainland China (2012–2015). The trial protocol (23) and primary outcomes (24) have been previously published. Exclusion criteria included: endocrine comorbidities; recent (past 2 months) use of hormonal/Chinese herbal therapies; peripartum status (delivery/miscarriage ≤6 weeks); active breastfeeding; missing metabolic data; and pregnancy loss to follow-up.

### Anthropometric measurements

At baseline, all participants received physical examinations that included anthropometric measurements (weight, height, waist circumference [WC], and hip circumference [HC]) and blood pressure assessment (systolic [SBP] and diastolic [DBP]). Body mass index (BMI) was calculated as weight in kilograms divided by height in meters squared (kg/m²), while waist-to-hip ratio (WHR) was derived from WC and HC measurements.

### Biochemical measurements

Fasting blood samples were collected at baseline following a 12-hour overnight fast. For regularly cycling women, samples were obtained on day 3 of the menstrual cycle, while women with amenorrhea provided samples on their enrollment day. All samples were analyzed at the core laboratory of Heilongjiang University of Chinese Medicine. The comprehensive biochemical analysis included: sex hormones and gonadotropins [total testosterone (TT), free testosterone (FT), sex hormone-binding globulin (SHBG), estradiol (E2), progesterone (P), luteinizing hormone (LH), follicle-stimulating hormone (FSH), and anti-Müllerian hormone (AMH)]; and metabolic parameters [fasting plasma glucose (FPG), fasting insulin (FIN), triglycerides (TG), total cholesterol (TC), high-density lipoprotein (HDL), low-density lipoprotein (LDL), lipoprotein A, apolipoprotein A1 (ApoA1), and apolipoprotein B (ApoB)]. Derived indices included: (a) free androgen index (FAI = TT [nmol/L]/SHBG [nmol/L] × 100); (b) triglyceride-glucose index (TyG = Ln[TG (mg/dL) × FPG (mg/dL)/2]); and (c) TyG-BMI (TyG × BMI). Conversion factors: FPG (1 mg/dL = 0.0555 mmol/L) and TG (1 mg/dL = 0.0113 mmol/L).

### Insulin resistance and metabolic syndrome

IR was defined as a HOMA-IR value ≥2.69 (25), calculated using the formula: HOMA-IR = FIN (mIU/L) × FPG (mol/L)/22.5 (26). MetS was diagnosed according to the Adult Treatment Panel III (ATP III) criteria (22), requiring the presence of at least three of the following five components: WC >88 cm; TG ≥150 mg/dL (1.7 mmol/L); HDL <50 mg/dL (1.3 mmol/L); blood pressure ≥130/85 mmHg; and FPG 100–126 mg/dL (5.6-6.9 mmol/L). For unit conversion, 1 mIU/L of FIN equals 6.965 pmol/L.

### Interventions

In the PCOSAct trial, participants were randomized (1:1:1:1 ratio) into four treatment arms: (a) clomiphene plus active acupuncture, (b) placebo plus active acupuncture, (c) clomiphene plus sham acupuncture, and (d) placebo plus sham acupuncture (n=250 per group). All groups underwent four consecutive treatment cycles, with pregnancy outcomes evaluated after each cycle. Upon achieving conception, interventions were discontinued while pregnancy outcomes were monitored through either miscarriage or live delivery.

### Fertility outcomes

Reproductive outcomes were defined as follows: ovulation - serum progesterone >5 ng/mL (15.9 nmol/L) during a treatment cycle; conception - serum hCG >10 IU/L (at which point all interventions ceased); clinical pregnancy - confirmed intrauterine gestation with fetal cardiac activity via transvaginal ultrasound; pregnancy loss - encompassing spontaneous abortion (<20 weeks), fetal death (≥20 weeks), and stillbirth (≥20 weeks); and live birth - delivery of a viable neonate at ≥20 weeks’ gestation.

### Statistical analyses

Statistical analyses were performed using SPSS 25.0 (IBM Corp.) and R Studio. Continuous variables were expressed as mean ± standard deviation or median (interquartile range), while categorical variables were presented as numbers (percentages). Intergroup comparisons were conducted using one-way ANOVA or Kruskal-Wallis test for continuous variables, and chi-square or Fisher’s exact test for categorical variables, as appropriate. Linear trend analyses were employed to examine the relationships between TyG-BMI quartiles and: anthropometric/biochemical parameters, prevalence of IR and MetS, and reproductive outcomes (ovulation and pregnancy). Binary logistic regression assessed associations between TyG-BMI and IR/MetS, while multinomial and ordinal logistic regression evaluated risk trends across TyG-BMI quartiles. The predictive performance of TyG-BMI for IR and MetS was determined using receiver operating characteristic (ROC) curve analysis, with calculation of the area under the curve (AUC), optimal cutoff values, sensitivity, specificity, and Youden index. Adjusted odds ratios (ORs) for outcomes were derived from logistic regression models incorporating covariates identified as significant in univariate analyses. Given the demonstrated effect of clomiphene (but not acupuncture) on live birth rates in PCOSAct, additional adjustment for clomiphene treatment was performed, and potential interactions between clomiphene and TyG-BMI were assessed using generalized linear models. The relationships between TyG-BMI and IR, MetS, and ovulation were investigated using restricted cubic spline (RCS) analysis, and likelihood ratio tests were performed. All tests were two-tailed, with P < 0.05 considered statistically significant.

## Results

Among the 1,000 women initially enrolled in PCOSAct, 145 were excluded due to missing glycolipid metabolic data or loss to follow-up, resulting in 855 PCOS patients included in the final analysis. The TyG-BMI was calculated for all participants and stratified into quartiles (Q1–Q4) with near-equal distribution (Q1: n = 214; Q2: n = 214; Q3: n = 213; Q4: n = 214).

### The anthropometric, biochemical characteristics, prevalence of IR, MetS and fertility outcomes of PCOS women across the quartiles of TyG - BMI

As presented in **Table 1**, all anthropometric parameters exhibited significant progressive increases across TyG-BMI quartiles (*P* and *P*-trend < 0.05 for all). Sex hormones demonstrated similar trends: FT and FAI levels rose with higher TyG-BMI quartiles, whereas E2, P, LH, LH/FSH, AMH, and SHBG showed inverse relationships (*P* and *P*-trend < 0.05). Metabolic parameters followed comparable patterns, with FPG, FIN, HOMA-IR, TC, TG, LDL, and ApoB displaying ascending trends, while HDL and ApoA1 progressively declined (*P* and *P*-trend < 0.05). The prevalence of both IR and MetS escalated markedly with increasing TyG-BMI quartiles (IR: Q1 = 7.94%, Q2 = 25.70%, Q3 = 54.46%, Q4 = 80.37%; MetS: Q1 = 0%, Q2 = 1.4%, Q3 = 11.74%, Q4 = 50.93%; *P* and *P*-trend < 0.05). Reproductive outcomes varied significantly among quartiles (*P* < 0.05 for ovulation, conception, and clinical pregnancy). Notably, the Q4 group exhibited higher ovulation and clinical pregnancy rates than Q1–Q2, and greater conception rates than Q1. Linear trend analyses revealed TyG-BMI was inversely associated with ovulation, conception, clinical pregnancy, and live birth, but positively correlated with pregnancy loss (*P*-trend < 0.05).

**Table 1:** Anthropometric, biochemical characteristics, prevalence of IR, MetS and fertility outcomes of the women with PCOS.

Characteristics	Q 1 N = 214	Q 2 N = 214	Q 3 N = 213	Q 4 N = 214	*P*-value	*P*-trend
Anthropometric characteristics						
Age(year)	27.31 ± 3.09	27.99 ± 3.14	28.10 ± 3.44	28.45 ± 3.43*	0.004	<0.001
BMI (kg/m^2^)	19.60(2.05)	22.31(1.94)*	25.04(2.04)* ^△^	28.89(4.26)* ^△#^	<0.001	<0.001
WC (cm)	74(9)	80.65(8)*	86(9.5)* ^△^	97(12.3)* ^△#^	<0.001	<0.001
HC (cm)	90.76 ± 5.68	95.486 ± 5.42*	99.96 ± 5.66* ^△^	107.06 ± 5.66* ^△#^	<0.001	<0.001
WHR	0.82(0.08)	0.85(0.07)*	0.87(0.07)* ^△^	0.91(0.07)* ^△#^	<0.001	<0.001
SBP (mmHg)	110(15)	110(10)*	110(10)*	120(10)* ^△#^	<0.001	<0.001
DBP (mmHg)	70(10)	73(10)	75(10)*	80(10)* ^△#^	<0.001	<0.001
Sex hormone profiles						
Estradiol (pmol/L)	207.4(121.2)	208.4(107.43)	195.5(104.2)	185.65(87.83)*	<0.001	<0.001
Progesterone (nmol/L)	1.90(1.29)	1.73(1.15)	1.74(1.21)	1.60(1.02)* ^△^	0.004	0.003
LH (mIU/mL)	11.29(9.36)	10.58(8.35)	9.62(8.18)	8.10(6.29)* ^△#^	<0.001	<0.001
FSH (mIU/mL)	6.25 ± 1.76	6.21 ± 1.60	5.87 ± 1.68*	6.01 ± 1.55	0.058	0.035
LH/FSH ratio	1.85(1.45)	1.71(1.32)	1.63(1.34)	1.30(0.95)* ^△#^	<0.001	<0.001
AMH (ng/mL)	12.79(9.77)	12.31(8.94)	11.07(7.67)*	9.46(8.76)* ^△^	<0.001	<0.001
Total testosterone (nmol/L)	1.53(0.91)	1.67(0.85)	1.58(0.85)	1.62(0.77)	0.475	0.829
Free testosterone (pg/ml)	2.12 ± 0.83	2.31 ± 0.87	2.28 ± 0.85	2.45 ± 0.77*	<0.001	<0.001
SHBG(nmol/L)	52.6(38.7)	41.5(32.78)*	28.2(21.85)* ^△^	21.95(12.95)* ^△#^	<0.001	<0.001
Free androgen Index	2.77(2.96)	4.00(4.06)*	5.50(5.52)*	7.36(5.62)* ^△#^	<0.001	<0.001
Glycolipid metabolic profiles						
Fasting glucose (mmol/L)	4.70(0.92)	4.95(0.95)*	5.17(0.88)* ^△^	5.34(1.08)* ^△^	<0.001	<0.001
Fasting insulin (pmol/L)	43.01(29.50)	63.03(45.21)*	86.39(58.16)* ^△^	129.25(80.47)* ^△#^	<0.001	<0.001
HOMA-IR	1.28(0.95)	1.96(1.55)*	2.80(2.09)* ^△^	4.53(3.11)* ^△#^	<0.001	<0.001
Cholesterol (mmol/L)	4.31(1.05)	4.68(1.33)*	4.77(1.28)*	5.02(1.57)* ^△^	<0.001	<0.001
Triglyceride (mmol/L)	0.88(0.43)	1.20(0.71)*	1.51(1.04)* ^△^	2.08(1.30)* ^△#^	<0.001	<0.001
TyG-BMI	161.76(18.88)	187.83(15.10)	218.72(15.72)	259.19(36.15)	<0.001	<0.001
High-density lipoprotein(mmol/L)	1.44(0.48)	1.29(0.46)*	1.16(0.44)* ^△^	1.10(0.39)* ^△^	<0.001	<0.001
Low-density lipoprotein(mmol/L)	2.55(0.96)	2.90(1.19)*	3.00(0.98)*	3.21(1.34)* ^△^	<0.001	<0.001
Lipoproteins A(mg/L)	97.15(77.73)	101.35(76.88)	102.20(80.95)	109.80(88.70)	0.509	0.586
Apolipoprotein A1 (g/L)	1.56(0.42)	1.51(0.41)*	1.41(0.46)	1.45(0.37)*	0.001	0.001
Apolipoprotein B (g/L)	0.69(0.25)	0.82(0.34)*	0.92(0.35)* ^△^	1.04(0.39)* ^△#^	<0.001	<0.001
IR(Yes)	17(7.94)	55(25.70)*	116(54.46)* ^△^	172(80.37)* ^△#^	<0.001	<0.001
MetS(Yes)	0	3(1.40)	25(11.74)* ^△^	109(50.93)* ^△#^	<0.001	<0.001
Fertility outcomes						
Ovulation	184(85.98)	186(86.92)	175(82.16)	156(72.90)* ^△^	<0.001	<0.001
Conception	81(37.85)	79(36.92)	78(36.62)	55(25.70)*	0.025	0.011
Clinical pregnancy	61(28.50)	58(27.10)	47(22.07)	34(15.89)* ^△^	0.008	0.001
Pregnancy loss	21/81(25.92)	24/79(30.38)	34/78(43.59)	21/55(38.18)	0.111	0.024
Live birth	55/81(67.90)	52/79(65.82)	40/78(51.28)	30/55(54.55)	0.078	0.023

IR, MetS, Ovulation and Pregnancy Outcomes n (%) are presented. Significant differences are denoted as follows: *P<0.05 vs. Q1 group; ^△^ *P*<0.05 vs. Q2 group; ^#^ *P*<0.05 vs. Q3 group.

### The correlation between TyG-BMI and IR, MetS in PCOS women

**Figure 1:**
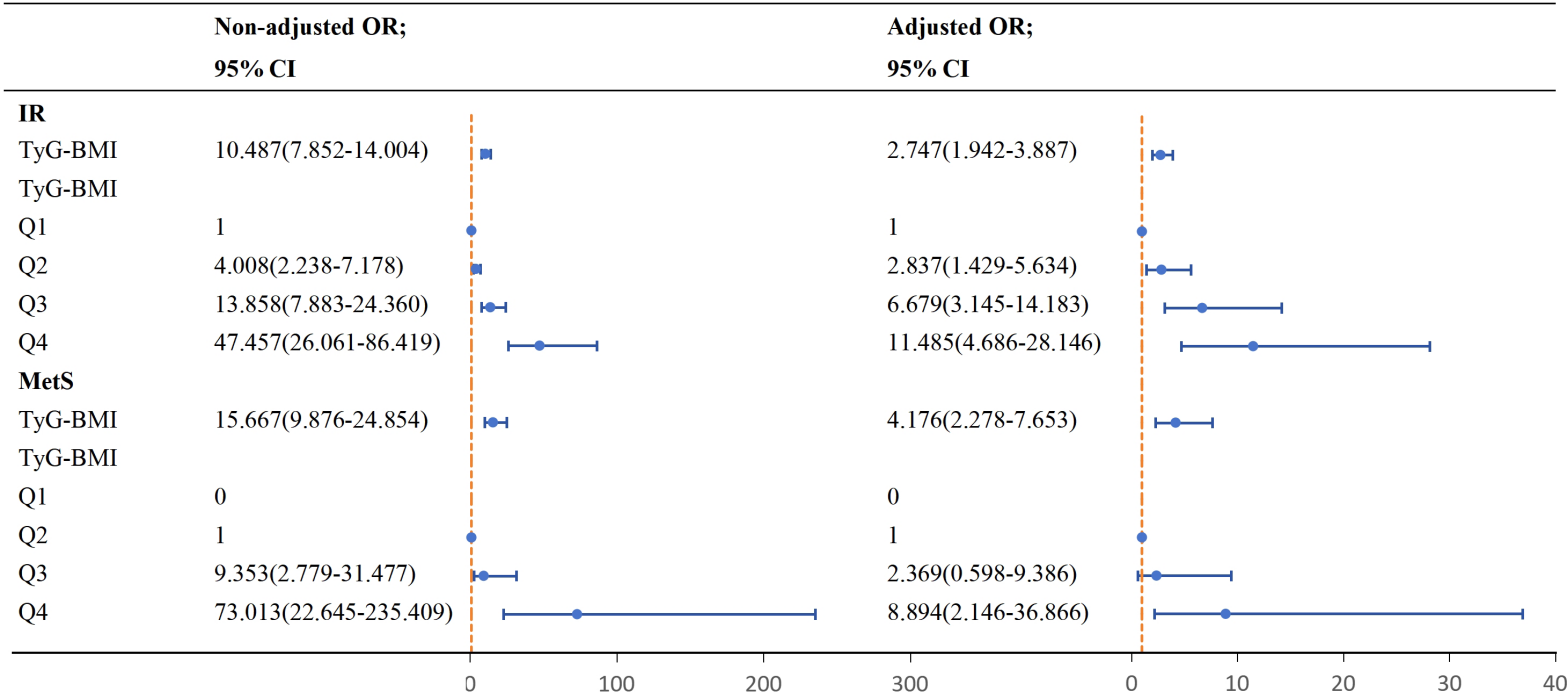
The correlation between TyG-BMI and IR, MetS in women with PCOS. IR: Adjusted for age, WC, HC, WHR, SBP, DBP, E2, P, LH, LF/FSH, AMH, FT, SHBG, FAI, TC, HDL, LDL, ApoA1, ApoB, MetS: Adjusted for age, WC, HC, WHR, SBP, DBP, E2, P, LH, LF/FSH, AMH, FT, SHBG, FAI, TC, HDL, LDL, ApoA1, ApoB, FIN, HOMA-IR, IR.

The TyG-BMI demonstrated significant associations with both IR and MetS (**Supplementary Table S1**). To further investigate these relationships, we performed multinomial and ordinal logistic regression analyses with adjustment for potential confounders (**Figure 1**). Ordinal logistic regression revealed that higher TyG-BMI quartiles were strongly associated with increased IR risk, with adjusted and unadjusted odds ratios (ORs) of 2.747 (95% CI: 1.942-3.887) and 10.487 (95% CI: 7.852-14.004), respectively. Notably, participants in the highest TyG-BMI quartile had a 10.485-fold increased risk of IR compared to those in the lowest quartile. Similarly, TyG-BMI showed a significant positive association with MetS. The adjusted OR for MetS was 4.176 (95% CI: 2.278-7.653), with the highest quartile exhibiting a 7.894-fold greater risk compared to the second quartile.

### Diagnostic performance of TyG-BMI for MetS, IR

**Figure 2:**
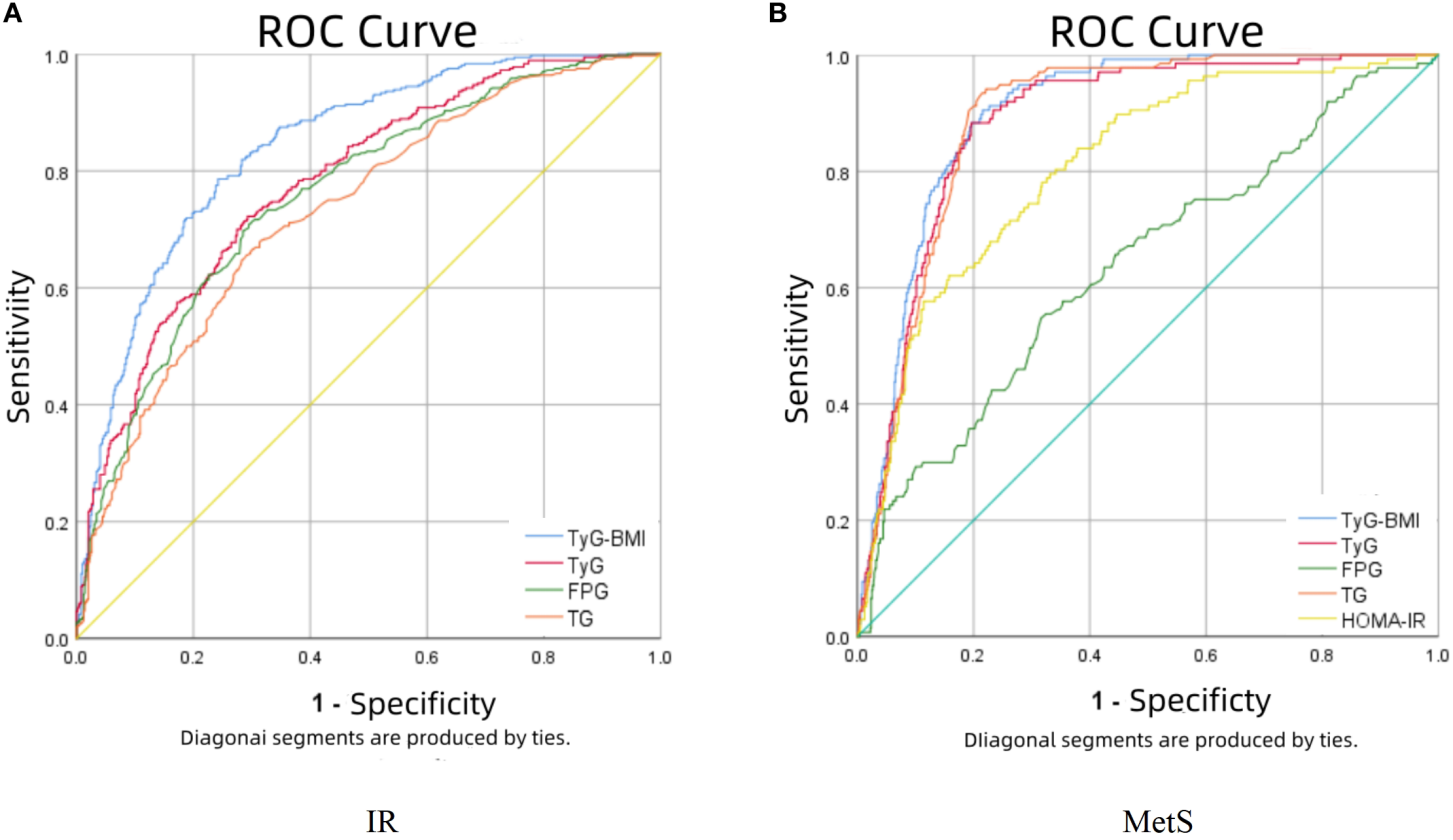
The results of ROC curve analysis regarding the predictability of TyG-BMI in IR **(A)** and MetS **(B)**.

**Figure 2** presents the predictive performance of TyG-BMI and other metabolic parameters for IR and MetS. For IR prediction (panel A), TyG-BMI demonstrated superior diagnostic accuracy among the four evaluated indicators, showing the largest area under the curve (AUC = 0.841) with an optimal cutoff value of 206.708 (sensitivity: 78.6%; specificity: 75.8%). Regarding MetS prediction (panel B), TyG-BMI, TyG, and TG levels showed overlapping ROC curves, though TyG-BMI maintained a marginal advantage (AUC = 0.899; sensitivity: 90.5%; specificity: 78.4%). Notably, TG exhibited the highest sensitivity (94.2%), while TyG showed the greatest specificity (80.4%) for MetS detection. Complete comparative data are provided in **Table 2**.

**Table 2:** TyG-BMI and other metabolic indicators predict AUC, cutoff value, sensitivity, and specificity prediction values for IR and MetS.

Outcomes	Variables	AUC(95%CI)	Cutoff value	Sensitivity	Specificty	You den Index
IR	TyG-BMI	0.841(0.815-0.867)	206.708	0.786	0.758	0.544
	TyG	0.781(0.750-0.811)	8.585	0.722	0.707	0.429
	Fasting glucose	0.759(0.727-0.792)	5.105	0.714	0.699	0.413
	triglyceride	0.732(0.698-0.765)	1.355	0.681	0.685	0.366
MetS	TyG-BMI	0.899(0.877-0.921)	225.104	0.905	0.784	0.689
	TyG	0.884(0.858-0.910)	8.903	0.883	0.804	0.687
	Fasting glucose	0.637(0.585-0.689)	5.315	0.547	0.684	0.231
	triglyceride	0.890(0.867-0.913)	1.695	0.942	0.779	0.721
	HOMA-IR	0.809(0.771-0.847)	2.773	0.796	0.669	0.465

### The correlation between TyG-BMI and fertility outcomes in PCOS women

Amongst 855 PCOS women, reproductive outcomes included ovulation (n=701, 82.0%), conception (n=293, 34.3%), clinical pregnancy (n=200, 23.4%), pregnancy loss (n=100, 34.1% of conceptions), and live birth (n=177, 20.7%). Analysis of TyG-BMI quartiles revealed significant treatment effects for clomiphene versus placebo across outcomes: for ovulation, odds ratios (ORs) were Q1 = 10.11 (95%CI 3.39-30.18), Q2 = 8.13 (2.71-24.36), Q3 = 8.00 (3.18-20.13), and Q4 = 3.34 (1.72-6.51) (**Supplementary Table S2**). Significant treatment differences were also observed for conception (Q2 OR = 2.48 [1.40-4.41]; Q3 OR = 3.70 [2.03-6.75]; Q4 OR = 3.59 [1.87-6.92]) and clinical pregnancy (Q2 OR = 2.16 [1.16-4.04]; Q3 OR = 2.66 [1.33-5.33]; Q4 OR = 3.36 [1.52-7.44]).

**Table 3** presents the association between TyG-BMI and fertility outcomes across progressively adjusted models. In the unadjusted Model 1, TyG-BMI demonstrated negative correlations with ovulation (OR: 0.992, 95% CI: 0.988-0.995), conception (OR: 0.995, 95% CI: 0.992-0.998), clinical pregnancy (OR: 0.993, 95% CI: 0.989-0.997), and live birth (OR: 0.993, 95% CI: 0.989-0.997), while showing a weak positive association with pregnancy loss (OR: 1.006, 95% CI: 1.000-1.012). After adjustment for clomiphene treatment and basic anthropometrics (Model 2), TyG-BMI remained significantly associated with ovulation (OR: 0.989, 95% CI: 0.981-0.997), conception (OR: 0.993, 95% CI: 0.987-0.999), and clinical pregnancy (OR: 0.992, 95% CI: 0.985-0.998). Further adjustment for sex hormones (Model 3) maintained the significant association with ovulation (OR: 0.990, 95% CI: 0.981-0.998). The fully adjusted Model 4 (including glycolipid metabolism parameters) continued to show TyG-BMI’s negative association with ovulation (OR: 0.984, 95% CI: 0.973-0.994). No significant interaction was observed between clomiphene and TyG-BMI for any fertility outcome (**Supplementary Table S3**).

**Table 3:** The correlation between TyG-BMI and ovulation, fertility outcomes in women with PCOS.

Fertility outcomes	Model 1	*P*-value	Model 2	*P*-value	Model 3	*P*-value	Model 4	*P*-value
	OR; 95% CI		OR; 95% CI		OR; 95% CI		OR; 95% CI	
Ovulation	0.992(0.988-0.995)	<0.001	0.989(0.981-0.997)	0.005	0.990(0.981-0.998)	0.015	0.984(0.973-0.994)	0.004
Conception	0.995(0.992-0.998)	0.002	0.993(0.987-0.999)	0.017	0.994(0.987-1.000)	0.051	0.995(0.988-1.003)	0.200
Clinical pregnancy	0.993(0.989-0.997)	<0.001	0.992(0.985-0.998)	0.016	0.994(0.987-1.001)	0.072	0.998(0.989-1.006)	0.606
Pregnancy loss	1.006(1.0-1.012)	0.043	1.002(0.992-1.013)	0.654	0.999(0.987-1.010)	0.807	0.987(0.973-1.002)	0.082
Live birth	0.993(0.989-0.997)	<0.001	0.995(0.988-1.002)	0.165	0.997(0.990-1.004)	0.421	1.002(0.993-1.011)	0.703

Model 1: Non-adjusted.

Model 2: Adjusted for clomiphene, age, WC, HC, WHR, SBP, DBP.

Model 3: Adjusted for clomiphene, age, WC, HC, WHR, SBP, DBP, E2, P, LH, LF/FSH, AMH, FT, SHBG, FAI.

Model 4: Adjusted for clomiphene, age, WC, HC, WHR, SBP, DBP, E2, P, LH, LF/FSH, AMH, FT, SHBG, FAI, TC, HDL, LDL, ApoA1, ApoB, FIN, HOMA-IR, IR.

### RCS analysis investigating the relationship between TyG-BMI and IR, MetS, and ovulation

**Figure 3:**
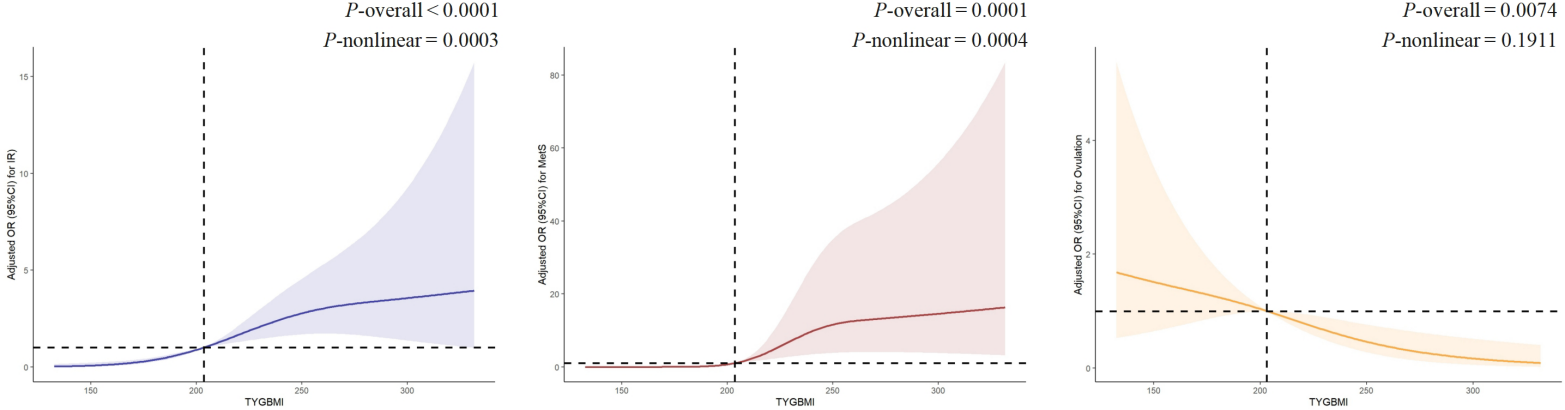
The RCS between TyG-BMI and IR, MetS and ovulation. IR: Adjusted for age, WC, HC, WHR, SBP, DBP, E2, P, LH, LF/FSH, AMH, FT, SHBG, FAI, TC, HDL, LDL, ApoA1, ApoB. MetS: Adjusted for age, WC, HC, WHR, SBP, DBP, E2, P, LH, LF/FSH, AMH, FT, SHBG, FAI, TC, HDL, LDL, ApoA1, ApoB, FIN, HOMA-IR, IR Ovulation: Adjusted for clomiphene, age, WC, HC, WHR, SBP, DBP, E2, P, LH, LF/FSH, AMH, FT, SHBG, FAI, TC, HDL, LDL, ApoA1, ApoB, FIN, HOMA-IR, IR.

Given the significant associations between TyG-BMI and IR, MetS, and ovulation, a piecewise regression analysis and RCS were performed (**Table 4**, **Figure 3**). The analysis identified a consistent inflection point at TyG-BMI = 203 for all three outcomes. Below this threshold, IR prevalence demonstrated a progressive increase with rising TyG-BMI values, reaching an odds ratio plateau beyond the inflection point. Notably, distinct patterns emerged post-inflection: while MetS prevalence continued to rise with increasing TyG-BMI, ovulation rates showed an inverse relationship. RCS analyses, after full covariate adjustment, revealed significant nonlinear associations between TyG-BMI and both IR (*P*-overall <0.001; *P*-nonlinear <0.001) and MetS (*P*-overall <0.001; P-nonlinear <0.001). In contrast, TyG-BMI exhibited a strictly linear correlation with ovulation status (*P*-overall <0.05; *P*-nonlinear >0.05). The likelihood ratio tests yielded statistically significant resulted for IR, MetS, and ovulation (*P*-overall <0.001).

**Table 4:** Threshold effect analysis between TyG-BMI and IR, MetS and ovulation.

Outcomes	TyG-BMI ≤ 203 N = 424		*P*-value	TyG-BMI > 203 N = 431		*P*-value	Likelihood ratio test
	Event no(%)	OR; 95% CI		Event no(%)	OR; 95% CI		
IR	56(13.2)	1.062(1.034-1.090)	<0.001	239(67.5)	1.014(1.001-1.027)	0.040	<0.001
MetS	3(0.7)	–	0.997	134(31.1)	1.020(1.006-1.033)	0.003	<0.001
Ovulation	367(86.6)	0.995(0.967-1.023)	0.720	334(77.4)	0.977(0.963-0.992)	0.002	<0.001

IR: Adjusted for age, WC, HC, WHR, SBP, DBP, E2, P, LH, LF/FSH, AMH, FT, SHBG, FAI, TC, HDL, LDL, ApoA1, ApoB.

MetS: Adjusted for age, WC, HC, WHR, SBP, DBP, E2, P, LH, LF/FSH, AMH, FT, SHBG, FAI, TC, HDL, LDL, ApoA1, ApoB, FIN, HOMA-IR, IR.

Ovulation: Adjusted for clomiphene, age, WC, HC, WHR, SBP, DBP, E2, P, LH, LF/FSH, AMH, FT, SHBG, FAI, TC, HDL, LDL, ApoA1, ApoB, FIN, HOMA-IR, IR.

## Discussion

In women with PCOS, we evaluated the association between TyG-BMI and multiple clinical parameters including circulating sex steroids, glucose/lipid metabolism, IR, MetS, and fertility outcomes. Our findings demonstrate that elevated TyG-BMI is significantly associated with more severe IR and MetS, establishing its value as a predictive marker for these metabolic disorders in PCOS patients. Additionally, we observed that higher TyG-BMI levels may impair ovulation, suggesting a potential role in reproductive dysfunction. Notably, these associations exhibited threshold effects: while IR prevalence plateaued beyond a certain TyG-BMI level, MetS prevalence continued to increase linearly, and ovulation rates showed a progressive decrease with rising TyG-BMI values.

Our findings establish TyG-BMI as a clinically valuable biomarker for identifying individuals at high risk of IR. The study demonstrates that both TyG and BMI independently correlate with IR, and their composite index (TyG-BMI) significantly enhances IR assessment by synergistically integrating adiposity-related metabolic effects (21). This improvement proves particularly relevant in obesity-prone populations, given obesity’s well-documented role in IR and metabolic dysfunction (27, 28). Clinically, TyG-BMI’s strong performance aligns with weight management strategies, which are fundamental to IR treatment, potentially guiding targeted interventions for obesity-related IR. ROC analyses confirmed TyG-BMI’s superior diagnostic accuracy over TyG alone in IR prediction. Notably, we observed that PCOS women with elevated BMI faced increased IR risk even when TyG levels were relatively low, highlighting BMI’s substantial contribution to IR development in this population. Furthermore, TyG-BMI demonstrated a dose-response relationship, with the effect potentially attenuating beyond a certain threshold point.

MetS represents a complex endocrine disorder characterized by multiple interrelated risk factors, including obesity (particularly visceral and abdominal adiposity), dyslipidemia, IR, hypertension, endothelial dysfunction, and systemic inflammation (29). While its precise pathogenesis remains incompletely understood, women with PCOS exhibit significantly elevated MetS risk (30), which is further exacerbated by concurrent overweight/obesity (31, 32). The TyG has demonstrated effectiveness in MetS identification (33), and visceral adiposity recognized is recognized as a key driver of MetS-related metabolic pathways (34). Our findings extend this understanding by revealing a positive correlation between increasing TyG-BMI levels and MetS risk progression, particularly above the inflection point. This observation supports the clinical rationale for combining BMI with TyG to enhance MetS prediction. This was evidenced by our results showing TyG-BMI’s superior diagnostic performance (AUC = 0.899 vs. TyG’s 0.884), with TyG-BMI offering better sensitivity while TyG maintained higher specificity.

This study demonstrates that elevated TyG-BMI is associated with poorer ovulation induction (more significant after the turning point), likely mediated through concurrent metabolic disturbances including obesity, IR, hyperandrogenism, and hyperlipidemia. Overweight/obesity frequently leads to ovulatory dysfunction - a primary infertility factor - through endocrine disruption and hypothalamic-pituitary- ovarian (HPO) axis dysregulation (35, 36). Specifically, obesity alters GnRH pulsatility and gonadotropin secretion. This occurs via increased peripheral androgen aromatization to estrogens, reduced SHBG, and elevated adipocyte leptin production. Concuruently, obesity-related IR exacerbates hyperandrogenemia (37, 38). These mechanisms are further supported by pregnant rat models showing androgen-induced uterine artery vasoconstriction and hypoxia-responsive gene upregulation (39). Our findings reveal that decreased SHBG levels with increased free testosterone and FAI correlate with elevated TyG-BMI. Weight gain exacerbates PCOS phenotype severity (40), and the obesity-PCOS synergy aggravates both metabolic and reproductive dysfunction (41), consistent with our observed TyG-BMI effects. IR plays a central role in PCOS-related lipid metabolism disorders by stimulating lipolysis and altering lipoprotein/hepatic lipase expression (40). The consequent dyslipidemia impairs oocyte quality and endometrial receptivity, partly through reduced adiponectin during implantation (42). Additionally, hyperinsulinemia directly compromises endometrial function and implantation efficacy (43), collectively contributing to the observed reproductive impairments. Although the association between TyG-BMI and ovulation is significant, its association with conception and live birth become non-significant in the adjusted models. This may be attributed to standardized ovulation protocols reducing the impact of metabolic factors on final delivery outcomes. Alternatively, male factors affecting embryo quality may have influenced conception and live birth rates.

This study possesses several notable strengths, including a large sample size representative of the Chinese PCOS population and comprehensively evaluating MetS, IR, and fertility outcomes through BMI-integrated assessment. Our findings suggest that TyG-BMI, a simple index derived from routine clinical measurements (triglycerides, fasting glucose, BMI), shows promise as a practical screening tool for identifying Chinese women with PCOS who are at heightened risk of significant insulin resistance and metabolic syndrome. A TyG-BMI value exceeding approximately 203 should prompt heightened clinical vigilance. This threshold may justify more intensive metabolic assessment and early intervention strategies, particularly those focused on lifestyle modification (weight control, dietary modification, and physical activity). Furthermore, the association between elevated TyG-BMI and reduced ovulation rates highlights the potential role of this integrated metabolic marker in assessing PCOS-related reproductive dysfunction. While further validation is needed, incorporating TyG-BMI assessment could help clinicians achieve a more comprehensive metabolic evaluation when managing infertility in this population.

However, certain limitations must be acknowledged. Firstly, as this is a secondary analysis of baseline data from the PCOSAct trial utilizing a cross-sectional design, our findings demonstrate associations but cannot establish causality between TyG-BMI and IR, MetS, or ovulation rates. The observed relationships may be bidirectional or influenced by unmeasured confounding factors. In the future, prospective longitudinal or interventional studies are needed to investigate whether changes in TyG-BMI cause alterations in metabolic or reproductive outcomes. Secondly, the absence of gold-standard IR diagnostic measures means our approach may not capture the full spectrum of insulin resistance dynamics. This is an important limitation affecting the precision of our primary outcome assessment related to insulin resistance. Thirdly, the lack of TyG-BMI measurements during second/third trimesters restrict pregnancy outcome analyses to baseline parameters, which limits the clinical applicability of findings regarding fertility outcomes. Consequently, the direct association between baseline TyG-BMI and long-term fertility success, including live birth rates, remains unknown and represents an important gap for future research. Furthermore, our findings are derived exclusively from a cohort of Chinese women with PCOS. Metabolic profiles, genetic factors, and PCOS phenotypic expression can vary significantly across different ethnicities and geographic regions. Therefore, the generalizability of our results—particularly the specific TyG-BMI threshold identified—to non-Chinese populations or other ethnicities with PCOS may be limited. This requires validation in diverse cohorts. Finally, due to incomplete data, potential confounding factors—such as dietary habits, physical activity levels, and medication use—were not adequately controlled for in the analysis. This limitation may affect the observed association between TyG-BMI and the study outcomes.

## Conclusion

In conclusion, our findings demonstrate that elevated TyG-BMI levels are significantly associated with IR, hyperlipidemia, and hyperandrogenism in Chinese women with PCOS. These results position TyG-BMI as a comprehensive and clinically reliable screening tool for both IR and MetS in this population. Furthermore, our data reveal that increased TyG-BMI may adversely affect ovulation induction outcomes. Notably, these associations show a threshold effect. Metabolic and reproductive impacts become particularly pronounced beyond specific TyG-BMI values.

## Data Availability

The raw data supporting the conclusions of this article will be made available by the authors, without undue reservation.
