# Supplementary material for: Triglyceride glucose index - body mass index predicts insulin resistance, metabolic syndrome and associates with impaired ovulation in Chinese women with polycystic ovary syndrome
Source: Front Endocrinol (Lausanne). 2025 Sep 18;16:1653636. doi: 10.3389/fendo.2025.1653636 (PMC12488453; doi:10.3389/fendo.2025.1653636)
Supplement: Supplementary file 1 [file Table1.docx]

| **Supplementary Table S 1 Significant variables associated with IR and MetS in women with PCOS .** | | | | | | | |
| --- | --- | --- | --- | --- | --- | --- | --- |
| **Outcomes** | **Variables** | **Variable in the equation** | | | | | |
|  |  | **B** | **S.E.** | **Wald** | **df** | **Sig.** | **Exp(B)** |
| **IR** | BMI | -1.068 | 0.182 | 34.259 | 1 | ＜0.001 | 0.344 |
|  | SHBG | -0.015 | 0.005 | 6.995 | 1 | 0.008 | 0.986 |
|  | triglyceride | -1.130 | 0.303 | 13.940 | 1 | ＜0.001 | 0.323 |
|  | TyG-BMI | 0.135 | 0.020 | 43.264 | 1 | ＜0.001 | 1.144 |
|  | Age | -0.074 | 0.029 | 6.749 | 1 | 0.009 | 0.928 |
| **MetS** | BMI | -2.435 | 0.476 | 26.130 | 1 | ＜0.001 | 0.088 |
|  | WC | -0.386 | 0.160 | 5.809 | 1 | 0.016 | 0.680 |
|  | HC | 0.503 | 0.153 | 10.785 | 1 | 0.001 | 1.654 |
|  | WHR*10 | 5.081 | 1.555 | 10.680 | 1 | 0.001 | 161.008 |
|  | FSH | -0.316 | 0.153 | 4.251 | 1 | 0.039 | 0.729 |
|  | total fasting glucose | -1.352 | 0.409 | 10.933 | 1 | ＜0.001 | 0.258 |
|  | total fasting insulin | 0.020 | 0.007 | 6.446 | 1 | 0.011 | 1.020 |
|  | triglyceride | -1.402 | 0.580 | 5.838 | 1 | 0.016 | 0.246 |
|  | TyG-BMI | 0.266 | 0.051 | 26.775 | 1 | ＜0.001 | 1.304 |
| All variable(s) entered: TyG-BMI, age, BMI, WC, HC, WHR, SBP, DBP, LH, FSH, LF/FSH, E2, P, TT, FT, SHBG, FAI, total fasting insulin and glucose, HOMA-IR, TC, TG, HDL, LDL, ApoA1, ApoB, lipoproteins A, clomiphene, acupuncture. Only significant variables are presented. | | | | | | | |

| **Supplementary Table S 2 Ovulation and pregnancy outcomes among the four interventions.** | | | | | |
| --- | --- | --- | --- | --- | --- |
| **Ovulation And Pregnancy outcomes** | **Q1 N=214** | **Q2 N=214** | **Q3 N=213** | **Q4 N=214** | ***P*-value** |
| **Ovulation** | 184/214(86.0) | 186/214(86.9) | 175/213(82.1) | 156/214(72.9)***^△^ | ＜0.001 |
| Active acupuncture plus clomiphene | 67/70(95.7) | 46/48(95.8) | 50/52(96.2) | 41/47(87.2) | 0.243 |
| Control acupuncture plus clomiphene | 45/46(97.8) | 61/63(96.8) | 55/59(93.2) | 43/52(82.7) | 0.011 |
| Active acupuncture plus clomiphene placebo | 32/50(64) | 39/48(81.3) | 37/52(71.2) | 34/53(64.2) | 0.2 |
| Control acupuncture plus clomiphene placebo | 40/48(83.3) | 40/55(72.7) | 33/50(66.0) | 38/62(61.3) | 0.075 |
| *P*-value^$^ | ＜0.001 | ＜0.001 | ＜0.001 | 0.003 |  |
| **Conception** | 81/214(37.9) | 79/214(36.9) | 78/213(36.6) | 55/214(25.7)*** | 0.025 |
| Active acupuncture plus clomiphene | 31/70(44.3) | 25/48(52.1) | 27/52(51.9) | 19/47(40.4) | 0.567 |
| Control acupuncture plus clomiphene | 22/46(47.8) | 27/63(42.8) | 29/59(49.1) | 19/52(36.5) | 0.549 |
| Active acupuncture plus clomiphene placebo | 13/50(26) | 16/48(33.3) | 8/52(15.4) | 9/53(17.0) | 0.112 |
| Control acupuncture plus clomiphene placebo | 15/48(31.3) | 11/55(20.0) | 14/50(28.0) | 8/62(12.9) | 0.091 |
| *P*-value^$^ | 0.070 | 0.005 | ＜0.001 | 0.001 |  |
| **Clinical pregnancy** | 61/214(28.5) | 58/214(27.1) | 47/213(22.0) | 34/214(15.9)***^△^ | 0.008 |
| Active acupuncture plus clomiphene | 22/70(31.4) | 19/48(39.6) | 15/52(28.8) | 12/47(25.5) | 0.493 |
| Control acupuncture plus clomiphene | 16/46(34.7) | 19/63(30.1) | 18/59(30.5) | 12/52(23.0) | 0.639 |
| Active acupuncture plus clomiphene placebo | 10/50(20.0) | 12/48(25) | 4/52(7.7) | 5/53(9.4) | 0.044 |
| Control acupuncture plus clomiphene placebo | 13/48(27.1) | 8/55(14.5) | 10/50(20) | 5/62(8.1) | 0.055 |
| *P*-value^$^ | 0.391 | 0.035 | 0.017 | 0.021 |  |
| **Pregnancy loss** | 21/81(25.9) | 24/79(30.4) | 34/78(43.6) | 21/55(38.2) | 0.111 |
| Active acupuncture plus clomiphene | 9/31(29.0) | 8/25(32) | 13/27(48.1) | 8/19(42.1) | 0.547 |
| Control acupuncture plus clomiphene | 6/22(27.2) | 8/27(29.6) | 12/29(41.3) | 6/19(31.5) | 0.734 |
| Active acupuncture plus clomiphene placebo | 4/13(30.7) | 4/16(25.0) | 5/8(62.5) | 3/9(33.3) | 0.354 |
| Control acupuncture plus clomiphene placebo | 2/15(13.3) | 4/11(36.3) | 4/14(28.5) | 4/8(50.0) | 0.286 |
| *P*-value^$^ | 0.594 | 0.944 | 0.441 | 0.916 |  |
| **Live birth** | 55/81(67.9) | 52/79(65.8) | 40/78(51.3) | 30/55(54.5) | 0.078 |
| Active acupuncture plus clomiphene | 18/31(58.1) | 17/25(68.0) | 12/27(44.4) | 11/19(57.9) | 0.378 |
| Control acupuncture plus clomiphene | 15/22(68.2) | 16/27(25.3) | 16/29(55.2) | 10/19(52.6) | 0.739 |
| Active acupuncture plus clomiphene placebo | 9/13(69.2) | 12/16(59.3) | 3/8(37.5) | 5/9(55.6) | 0.325 |
| Control acupuncture plus clomiphene placebo | 13/15(86.7) | 7/11(63.6) | 9/14(62.3) | 4/8(50) | 0.308 |
| *P*-value^$^ | 0.356 | 0.762 | 0.562 | 0.980 |  |
| *Note*: n (%) are presented.Significant differences are denoted as follows: **P<0.05* vs. Q1 group; ^△^*P*<0.05 vs. Q2 group; ^#^*P*<0.05 vs. Q3 group. *P*-value^$^ of four intervention comparison within group. | | | | | |

| **Supplementary Table S 3 The Interaction between clomiphene and TyG-BMI in fertility outcomes.** | | | |
| --- | --- | --- | --- |
| **Outcomes** | **Wald** | **df** | **Sig.** |
| Ovulation | 6.697 | 3 | 0.082 |
| Conception | 1.782 | 3 | 0.618 |
| Clinical pregnancy | 1.983 | 3 | 0.576 |
| Pregnancy loss | 1.014 | 3 | 0.798 |
| Live birth | 1.601 | 3 | 0.659 |
| *Note*: Adjusted for Model 4. | | | |
